# Multigene analysis reveals ‘*Candidatus* Phytoplasma asiaticum’ (16SrII-C) association with niger and sesame phyllody in Madhya Pradesh, Central India and identification of a putative vector

**DOI:** 10.3389/fpls.2026.1768105

**Published:** 2026-04-13

**Authors:** M. Gurivi Reddy, K. N. Gupta, Shivani Gupta, A. K. Vishwakarma, Rashmi Yadav, Ravinder Kumar, Rasna Maurya, Govind P. Rao

**Affiliations:** 1 Department of Plant Pathology, Agricultural College, Acharya N.G. Ranga Agricultural University, Mahanandi, Andhra Pradesh, India; 2 Project Coordinating Unit, AICRP on Sesame and Niger, ICAR, Jawaharlal Nehru Krishi Vishwa Vidyalaya (JKNVV), Jabalpur, Madhya Pradesh, India; 3 Division of Plant Pathology, Indian Agricultural Research Institute, New Delhi, India; 4 Division of Germplasm Evaluation, ICAR – National Bureau of Plant Genetic Resources, New Delhi, India

**Keywords:** 16S rRNA, 16SrII-C subgroup, niger, PCR assay, phyllody, *sec*A, *sec*Y, sesame

## Abstract

A field investigation was conducted during the winter seasons of 2021–22 and 2022–23 at the Jawaharlal Nehru Krishi Vishwa Vidyalaya (JNKVV), Jabalpur, Central India, to identify and characterize the phytoplasma strains associated with niger (var. JNC6) and sesame (var. RT315) exhibiting severe phyllody symptoms. Disease incidence ranged from 0-3%, and 3 to 15% were recorded in niger and sesame crops during the study period. Nested PCR assays using universal phytoplasma primer pairs P1/P7 followed by R16F2n/R16R2 consistently amplified ~1.25 kb fragments of the 16S rRNA gene from both the symptomatic plants, confirming phytoplasma association. Additional amplification of phytoplasma-specific *sec*A (~600 bp) and *sec*Y (~1.7 kb) gene fragments further validated the phytoplasma infection in both crops. Two predominant leafhopper species, *Amrasca biguttula* and *Orosius albicinctus*, colonizing and feeding niger and sesame fields were identified. Notably, only *Amrasca biguttula* was tested positive for phytoplasma using the similar PCR assays mentioned above, implicating it as a putative vector. Sequence identity, phylogenetic analysis, and *in silico* RFLP profiling of 16S rRNA, *sec*A, and *sec*Y gene sequences from both the symptomatic plants and leafhoppers confirmed the presence of a ‘*Ca.* P. asiaticum’ related strain (16SrII-C subgroup). This study represents the first global report of 16SrII-C subgroup phytoplasma infection in niger, highlighting a new host record. The detection of an identical phytoplasma strain in niger, sesame, and *Amrasca biguttula* suggests a possible epidemiological link and underscores the role of these natural hosts and the leafhopper in the dissemination of ‘*Ca.* P. asiaticum’-related phytoplasma strains in Central India.

## Introduction

Phytoplasmas are wall-less, phloem-limited bacteria that belong to the class Mollicutes (Lee et al., 1998). Since they cannot be cultured in cell-free media, their taxonomy and identification rely on molecular characterization and gene sequence analysis. Currently, 48 ‘*Candidatus* Phytoplasma’ species belonging to 37 groups and over 150 subgroups have been identified on over 800 plant species (Bertaccini et al., 2022; Wei and Zhao, 2022). Many phytoplasma strains have been identified associated with newly emerging plant diseases worldwide in recent years (Rao et al., 2018; Huang et al., 2023; Tiwari et al., 2023). They are naturally transmitted by sap sucking insects belonging to the order Hemiptera, mainly in the families Cicadellidae, Cixiidae and Psyllidae (Weintraub and Beanland, 2006). Besides oilseeds, brassicas, peanut, sesame, linseed (flax), sunflower and soybean have been reported as hosts of different phytoplasma strains (Rao et al., 2018; Vemana et al., 2023). Niger, scientifically known as *Guizotia abyssinica* (L. f.), family Asteraceae, is a vital oilseed crop. Its seeds contain 32-40% oil and 18-24% protein (Ranganatha, 2014), making it a valuable resource for food and nutritional security, particularly in tribal regions (Getinet and Sharma, 1996). Native to Ethiopia, niger is cultivated in various countries, including South Africa, East Africa, West Indies, Zimbabwe, Uganda, Iran, and India (Papade et al., 2025). However, India leads the world in niger production, area, and export, with major cultivation in tribal areas of Madhya Pradesh, Orissa, Maharashtra, Gujarat, Bihar, Karnataka, and Andhra Pradesh (Papade et al., 2025).

Niger crop plays a significant role in soil conservation, land rehabilitation, and as a green manure crop, enhancing the yield of subsequent crops (Ranganatha, 2014). However, its production is threatened by insect pests, diseases and weeds. The important diseases of niger are Ozonium wilt (*Ozonium texanum* var. *parasiticum* Thirum.), collar rot (*Sclerotium rolfsii* Sacc.), *Macrophomina* root and stem rot [*Macrophomina phaseolina* (Maubl.) Ashby], damping off/root rot (*Rhizoctonia solani*), leaf spots (*Cercospora guizoticola*, *Alternaria* sp., *Curvularia lunata*), powdery mildew (*Sphaerotheca* sp.), rust (*Puccinia guizotiae*) and bacterial leaf spot (*Xanthomonas campestris* pv. *guizotiae*) (Gupta et al., 2018; Kolte, 1985).

Phytoplasma association has also been described earlier on the niger crop from India and abroad. Earlier occurrence of the aster yellows group (16SrI-B) has been reported from South India (Muttappagol et al., 2022). Besides, ‘*Ca*. P. asteris’ and 16SrII-D subgroup have been reported to be associated with niger phyllody from Iran (Muttappagol et al., 2022). Consistent occurrence of leaf phyllody symptoms was noticed in the niger variety JNC6 at experimental fields of JNKVV, Jabalpur University campus, Madhya Pradesh, which causes serious yield loss to the niger crop every year. But no detailed study has been performed to record the etiology of the phyllody disease. Gupta et al. (2023) only suspected phytoplasma association in niger crop based on symptoms, but phytoplasma etiology has never been confirmed in India on niger crops. Hence, in the present investigation, an attempt was made to investigate the multigene characterization of a phytoplasma strain associated with niger and sesame phyllody at Jabalpur, Madhya Pradesh province of India.

In addition to niger, sesame (*Sesamum indicum* L.) is an ancient oilseed crop, domesticated over 3,000 years ago, belonging to the Pedaliaceae family. The demand for sesame seeds has increased in the last two decades due to high oil quality, protein content, antioxidant content, and wide adaptability in extreme climatic and edaphic environments (Myint et al., 2020; Moazzami and Kamal-Eldin, 2006). It’s cultivated in tropical and sub-tropical areas of Asia and Africa, covering 40°N to 40°S latitude. Sesame is a significant oilseed crop, with 6.5 million hectares cultivated worldwide, producing over 3 million tons of seed. Major producers include India, Sudan, Myanmar, and China, accounting for 68% of global production (Yadav et al., 2022). Sesame seed is rich in protein (20%) and edible oil (50%), with high amounts of saturated fatty acids (47% oleic acid and 39% linolenic acid) (Uzun et al., 2008). Among various biotic stresses, phyllody is one of the more destructive diseases of sesame caused by phytoplasmas and causes significant yield losses from 34 to 100% in Asian and African countries (Rao and Nabi, 2015; Yadav et al., 2022). Phytoplasmas belonging to the 16SrII group, particularly the subgroup 16SrII-C, are commonly associated with phyllody and witches’ broom symptoms in several crops across Asia and the Middle East. ‘*Ca.* P. asiaticum,’ the causal agent of witches’ broom disease of lime, represents one of the most economically important members of this group and is known for its wide host range and genetic heterogeneity. Previous studies have demonstrated considerable diversity within 16SrII strains using multigene analyses targeting housekeeping and secretory pathway genes such as *sec*A, *sec*Y, *tuf*, and *rp*. However, the genetic architecture and diversity of 16SrII-C strains infecting oilseed crops like niger and sesame in India remain sparsely characterized, limiting accurate classification and epidemiological interpretation. A wide genetic diversity was identified among sesame phyllody-associated phytoplasmas and four ribosomal groups, 16SrI, 16SrII, 16SrVI and 16SrIX were detected (Rao and Nabi, 2015; Yadav et al., 2022). Understanding phytoplasma epidemiology requires clear identification of insect vectors, primarily leafhoppers and planthoppers that transmit the pathogen in a persistent and circulative manner.

Although several phytoplasmas within the 16SrII group are known to be transmitted by diverse Hemipteran species, the specific vectors responsible for transmission of 16SrII-C (*Ca*. P. asiaticum) strains affecting niger and sesame in central India remain largely unknown. The lack of molecular confirmation of phytoplasma presence in local insect populations poses a major challenge to the development of effective disease management strategies. Identifying putative vectors is therefore a critical step toward elucidating the transmission pathways and devising targeted vector-control interventions. The disease spreads in nature by different leafhopper species and many weed species are reported hosts of the sesame phyllody-associated phytoplasmas. No effective control measures of the disease were developed, except resistance, management of insect vectors and altering the dates of sowing to avoid peaks of insect vector population. So far, no resistant genotype of sesame against sesame phyllody is available (Yadav et al., 2022).

The present study aimed to (i) detect and characterize phytoplasma strains associated with phyllody disease in niger and sesame grown in Jabalpur, Madhya Pradesh, (ii) perform multigene-based phylogenetic and *in silico* analyses to confirm their identity as ‘*Ca.* P. asiaticum’ (16SrII-C), and (iii) identify potential insect vectors by surveying local leafhopper and planthopper populations and detecting phytoplasma DNA in candidate species. This integrated approach provides new insights into the genetic diversity and epidemiology of 16SrII phytoplasmas in central India and contributes to the foundational knowledge required for developing systematic disease management strategies.

## Material and methods

### Survey and collection of plant samples

A roving survey was conducted in university experimental fields at Jawaharlal Nehru Krishi Vishwa Vidyalaya (JNKVV) campus in Jabalpur, Madhya Pradesh, India, during October-November of the 2021-22 to 22-23 cropping seasons. The region falls under a tropical monsoon climate, with annual rainfall of 900–1,300 mm and temperatures ranging from 10 °C (winter) to 42 °C (summer). Surveys were carried out in the niger and sesame fields in Madhya Pradesh. A plot area of 5 x 5m was selected randomly in the niger and sesame fields and the total number of healthy and symptomatic plants showing phyllody symptom was recorded as per visual observations and the per cent disease incidence was calculated by averaging the incidence of three spots (5x5m) in each field by using the formula.


$$\text{Percent disease incidence }=\;\frac{\text{No. of plants infected}}{\text{Total no. of plants}}\;\times \;100$$

The mean per cent of disease incidence was calculated in each individual field/experimental plot.

Ten symptomatic and asymptomatic niger and sesame leaf samples were collected from the fields and glasshouse (for healthy samples), respectively. All the collected plant samples were packed in polythene bags and kept in a deep freezer at -80 °C for PCR analysis at the Division of Plant Pathology, Indian Agricultural Research Institute, New Delhi for PCR assays.

### Insect vector sampling and molecular characterization

During the field survey, different leafhopper species feeding on niger and sesame plants at JNKVV experimental fields of niger and sesame crops were collected using the sweeping net method during morning hours in October months of 2021-22 and 22-23. The collected insects were kept in plastic vials containing 70% ethanol and stored at 4 °C and identified at the Division of Entomology, ICAR-Indian Agricultural Research Institute, New Delhi. The identified insect samples belonging to leafhopper species were used for phytoplasma indexing. To monitor the insect vector population, yellow sticky trap cards @ 1-2 traps per 50 sq mt (placed at East, West, South and North side of the field, two meters inside the border row at a height of 40 cm near to the crop canopy) were fixed in the 4-6 weeks old niger (variety JNC6) and sesame (variety RT351) fields at JNKVV, Jabalpur. Leaf hopper population sticked on yellow cards was counted after a week interval for two months in two consecutive years (2022 and 2023) at a weekly interval in the months of October-November and correlated with the observational trends of niger and sesame phyllody disease incidence in the same fields.

### DNA isolation from plant and insect samples

Total genomic DNA was isolated from the symptomatic and asymptomatic niger and sesame leaf samples using Qiagen DNeasy plant mini kit (Germany) and from the insect using the Qiagen DNeasy Blood and Tissue kit (Germany) as per the manufacturer’s protocol from different surveyed locations. Extracted DNA was quantified using NanoDrop spectrophotometry, with concentrations ranging from 100-120 ng/µL and A260/280 purity ratios of 1.8-1.9, confirming high-quality, protein-free DNA suitable for downstream PCR applications. Samples meeting these established thresholds—indicative of pure nucleic acids with minimal contaminants—were diluted to 50 ng/µL and selected as templates to ensure reliable PCR amplification and trustworthy negative results.

### Identification of phytoplasma by PCR assays

The extracted DNA from niger, sesame and insect was amplified for 16S ribosomal DNA with phytoplasma-specific universal primer pair P1/P7 (Deng and Hiruki, 1991; Schneider et al., 1995), followed by nested primer pair R16F2n/R16R2 (Gundersen and Lee, 1996) (**Table 1**) making a final volume of 25 μl comprising of 12.5 μl of OnePCR ™ 2X PCR Master Mix (GeneDireX, Taiwan), 0.5 μl of each forward and reverse primer 10 pmol/μl (final concentration of 0.2 μM), 10.5 μl of nuclease-free water (Sisco Research Laboratories Pvt. Ltd., India) and 1 μl of DNA template (=50 ng). All the PCR reactions were carried out in a thermal cycler (Mastercycler, Eppendorf, Hamsburg, Germany). The direct PCR assay using the primer pair P1/P7 was performed with the PCR conditions: 94 °C for 5 minutes for the initial denaturation step followed by 35 cycles of 94 °C for 45 seconds of denaturation; 55 °C for 1 minute of annealing; 72 °C for 2 minutes of extension and 72 °C for 10 minutes of final extension. The products obtained from the amplification of the direct PCR were diluted (1:30) with nuclease free water and 2 μl of the diluted product was used as template in nested PCR assays using the primer pair R16F2n/R16R2, keeping all the PCR conditions the same except for the annealing temperature which was raised to 56 °C.

**Table 1:** Phytoplasma universal/group specific primers used for conventional PCR assays in study.

Primer name	Sequence	Target gene	Amplicon size (bp)	References
P1	5’ AAG AGT TTG ATC CTG GCT CAGGAT T 3’	*16SrRNA*	1800	Deng and Hiruki, 1991; Schneider et al., 1995
P7	5’CGT CCT TCA TCG GCT CTT 3’			
R16F2n	5’GAA ACG ACT GCT AAG ACT GG3’	*16SrRNA*	1250	Gundersen and Lee, 1996
R16R2	5’ TGA CGG GCG GTG TGT ACA AAC CCC G 3’			
*sec*Afor1	5’AAGAGTTTGATCCTGGCTCAGGATT 3′	*sec*A	840	Hodgetts et al., 2008
*sec*Arev3	5′CGTCCTTCATCGGCTCTT 3′			
*sec*Afor5	5′ GATGAGGCTAGAACGCCT 3′	*sec*A	600	Bekele et al., 2011
*sec*Arev2	5′ GAAACGACTGCTAAGACTGG 3′			
*sec*YF1	5′ CAGCCATTTTAGCAGTTGGTGG 3′	*secY*	1700	Lee et al., 2010
*sec*YR1	5′ CAGAAGCTTGAGTGCCTTTACC 3′			
*sec*YF2	5′ TGAAGGTGGTCAAACTCCT 3′	*secY*	1700	
*sec*YR1	5′ CAGAAGCTTGAGTGCCTTTACC 3′			

Amplification of two multilocus candidate genes (*sec*A and *sec*Y) was also employed by the utilization of primer pairs: *sec*A (secAfor1/secArev3 followed by nested PCR primers SecAfor5/SecArev2) (Hodgetts et al., 2008; Bekele et al., 2011), and *sec*Y (SecYF1(II)/SecYR1(II) followed by semi-nested PCR primers SecYF2 (II)/SecYR1 (II) (Lee et al., 2010) (**Table 1**).

### Nucleotide sequencing

Two amplified *16S rRNA*, *sec*A and *sec*Y gene fragments from niger, sesame and insect samples analyzed in the study were purified with the Wizard^®^ SV Gel and PCR Clean-Up System (Promega, Madison, USA). The purified PCR products of the 16S rRNA, *sec*A and *sec*Y genes were ligated into the pGEM^®^-T vector (Promega, Madison, USA) and cloned into competent *Escherichia coli* (DH5-α) cells. At least two recombinant clones of PCR amplified products were sequenced directly in both directions using the same set of primers as for the PCR amplification at Eurofins Genomics Pvt. Ltd., Karnataka. Nucleotide sequences of each amplicon were assembled using the Contig Assembling Program function, trimmed to the annealing sites of the primers R16F2n/R16R2, and aligned to obtain a consensus sequence in the software BioEdit, version 7.1.3.0. The pair-wise sequence comparison analysis was done through BLAST analysis. The original forward and reverse sequence data of each test sample were edited, aligned and assembled with CLC Genomics Workbench 12.0 (https://www.qiagenbioinformatics.com/) and sequences of the representative strains were deposited in GenBank (NCBI, Bethesda, MD, USA) data library and accession numbers were received.

### Phylogenetic analysis

Nucleotide sequences of different representative phytoplasma groups were retrieved from GenBank and were aligned with phytoplasma sequences available in GenBank (**Supplementary Table 1**) using CLC Genomics Workbench 12.0 (https://www.qiagenbioinformatics.com). Phylogenetic trees were constructed using the neighbor-joining method for phytoplasma with MEGA 11.0 software (Kumar et al., 2016) using 1000 bootstrap replications. Sequences of *Acholeplasma laidlawii* (Acc. no. AB680603) were used as an outgroup to root the phylogenetic trees of 16S ribosomal gene and *A. oculi* (Acc. no. LK028559) to root the phylogenetic trees of the *sec*A and *sec*Y genes.

### Virtual RFLP analysis

Virtual RFLP analysis was carried out for R16F2n/R16R2 fragments of 16Sr RNA gene derived from identified phytoplasma strains from plants and insects and were submitted to *iPhy*Classifier online tool (Zhao et al., 2009). The different restriction profiles, obtained with 17 restriction endonucleases (*Bam*HI, *Bfa*I, *Alu*I, *Bst*UI, *Hae*III, *Eco*RI, *Dra*I, *Hin*fI, *Hpa*II, *Hha*I, *HpaI*, *Kpn*I, *Sau3*AI, *Ssp*I, *Rsa*I, *Mse*I, *Taq*I) of different phytoplasma isolates in virtual gel plotting, were compared with the virtual RFLP pattern from the standard representative group/subgroup reference strains of phytoplasma by the same restriction enzymes and similarity coefficient values. A similarity coefficient (F) was calculated for each pair of phytoplasma strains according to the formula, F = 2Nxy/(Nx +Ny), in which x and y are two given strains under investigation, Nx and Ny are the total number of DNA fragments (bands) resulting from digestions by 17 enzymes for strains x and y, respectively, and Nxy is the number of fragments shared by the two strains.

## Results

### Field symptoms and disease incidence

**Figure 1:**
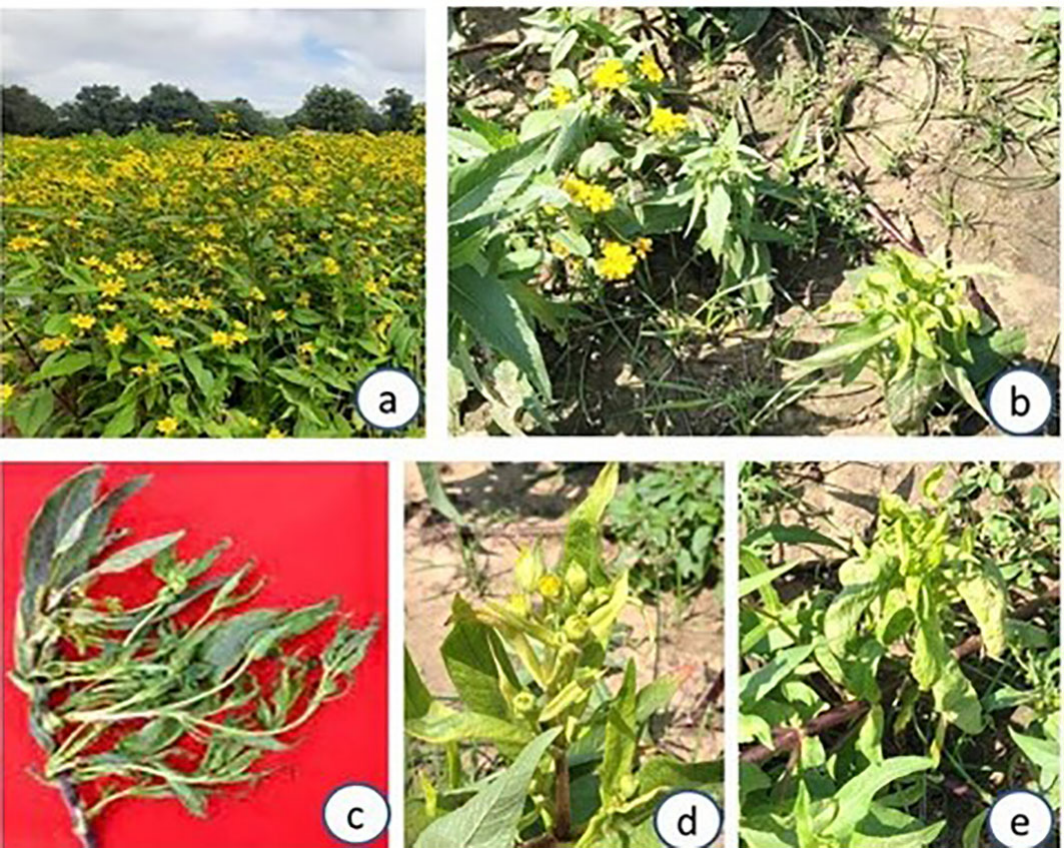
Phytoplasma-associated symptoms on Niger plant fields at JNKVV, Jabapur, on var JNC 6; **(a)** healthy crop; **(b)** healthy and symptomatic plants with phyllody symptoms and stunting; **(c)** severe phyllody symptoms with young shoot proliferation; **(d, e)** leaf yellowing and phyllody symptoms with malformed flower buds.

**Figure 2:**
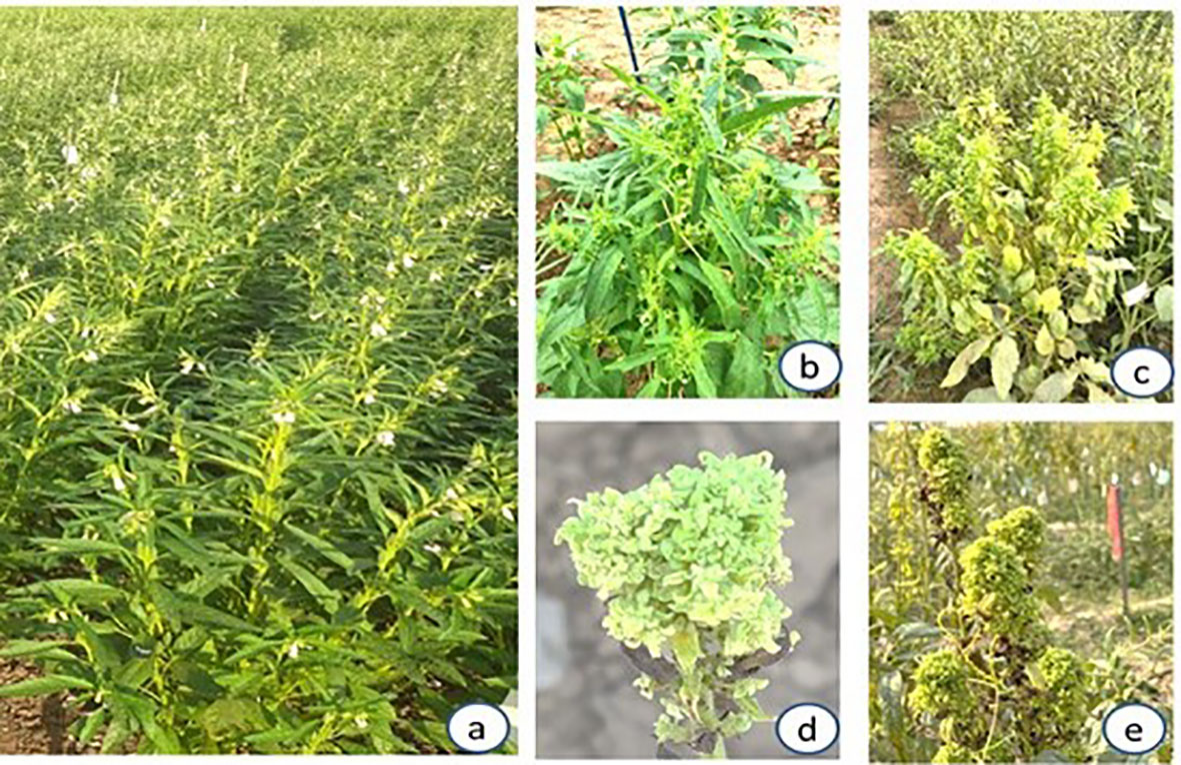
Phytoplasma-associated symptoms on sesame at JNKVV, Jabalpur; **(a)** healthy sesame crop; **(b)** little leaf; **(c)** witches’ broom symptoms; **(d, e)** severe phyllody and witches’ broom symptoms.

Survey of niger and sesame fields in JNKVV campus of Jabalpur during 2021-22 to 22-23 season revealed a widespread occurrence of niger phyllody and leaf yellowing disease in niger fields of variety JNC6. At later stages shoot proliferation and flower bud malformation symptoms were also recorded (**Figure 1**). Besides niger, severe symptoms of phyllody and witches’ broom were recorded in sesame crop (variety RT351) grown in nearby niger fields in the same university campus (**Figure 2**). An average disease incidence ranging from 0-3% in different niger fields and 3-15% in sesame fields was observed on the basis of visual observation of symptoms over asymptomatic plants during 2021-22 and 2022-23 season, respectively (**Table 2**).

**Table 2:** Identification of phytoplasma associated with niger, sesame and insect in the present study.

Hosts	Symptoms	Isolates	Incidence (%)*		GenBank accession numbers for the genes		
			2021–2022	2023–2024	16S rRNA	*sec*A	*sec*Y
*Niger*	Phyllody and flower malformation	JB-5	0.0	2.4	PX636087	PX531336	PX531330
		JB-6	1.2	3.0	PX636088	PX531337	PX531331
*Sesame*	Phyllody and a witches’ broom	JB-1	4.6	9.8	PX636085	PX531334	PX531328
		JB-2	6.0	15.0	PX636086	PX531335	PX531329
*Amrasca biguttula*		JB-9	–	–	PX636089	PX531338	PX531332
		JB-10	–	–	PX636090	PX531339	PX531333

*Average incidence in three plot size of 5X5 m.

### Leafhopper identification and population studies

**Figure 3:**
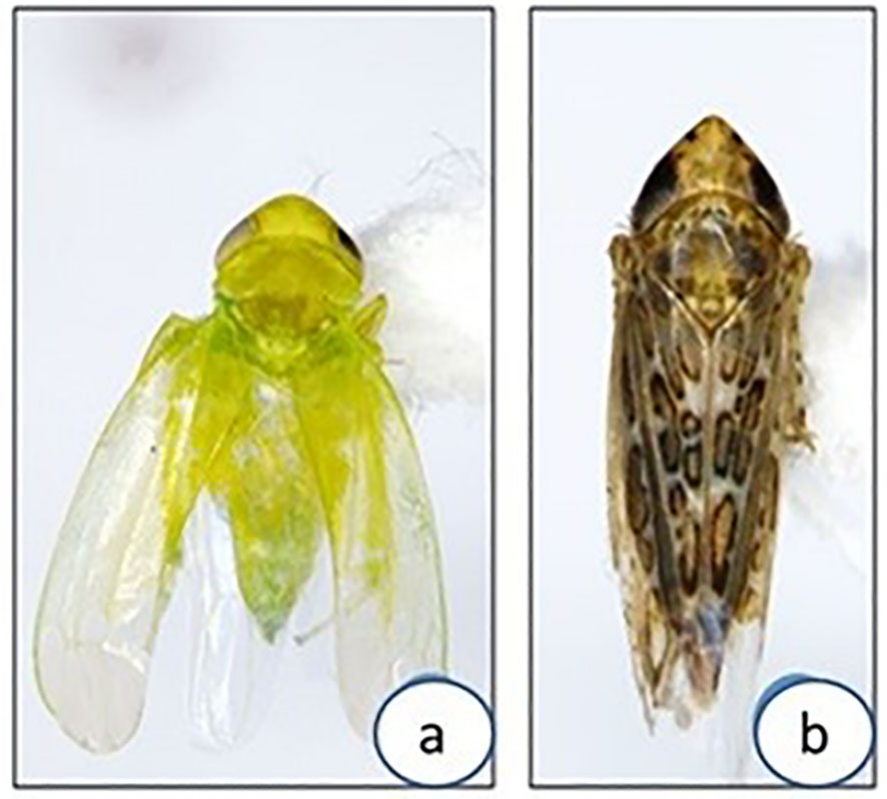
Leafhoppers morphology under microscope **(a)** *Amrasca biguttula*, **(b)** *Orosius albicinctus* collected from sesame and niger fields in the study.

Two major leafhopper species collected from niger and sesame fields at experimental plots were identified as *Amrasca biguttula* (Ishida) and *Orososius albicinctus* (Distant). *Amrasca bigutulla* was identified as the predominant species present in the niger and sesame fields in both the survey years at JNKVV, Jabalpur (**Figure 3**). The highest count of insect vector (*Amrasca biguttula*) was recorded during the second fortnight of October 2023 (35 insects/trap) to the first fortnight of November (22 insects/trap). However, *O. albicinctus* was counted less in number during the second fortnight of October 2023 (12 insects/trap) than in the first fortnight of November (05 insects/trap). The high populations of *Amrasca biguttula* in experimental fields of niger and sesame were suggested to be positively correlated observational trends with the incidence of niger and sesame phyllody in later months.

### Molecular detection of phytoplasma associated with niger and sesame crops

**Figure 4:**
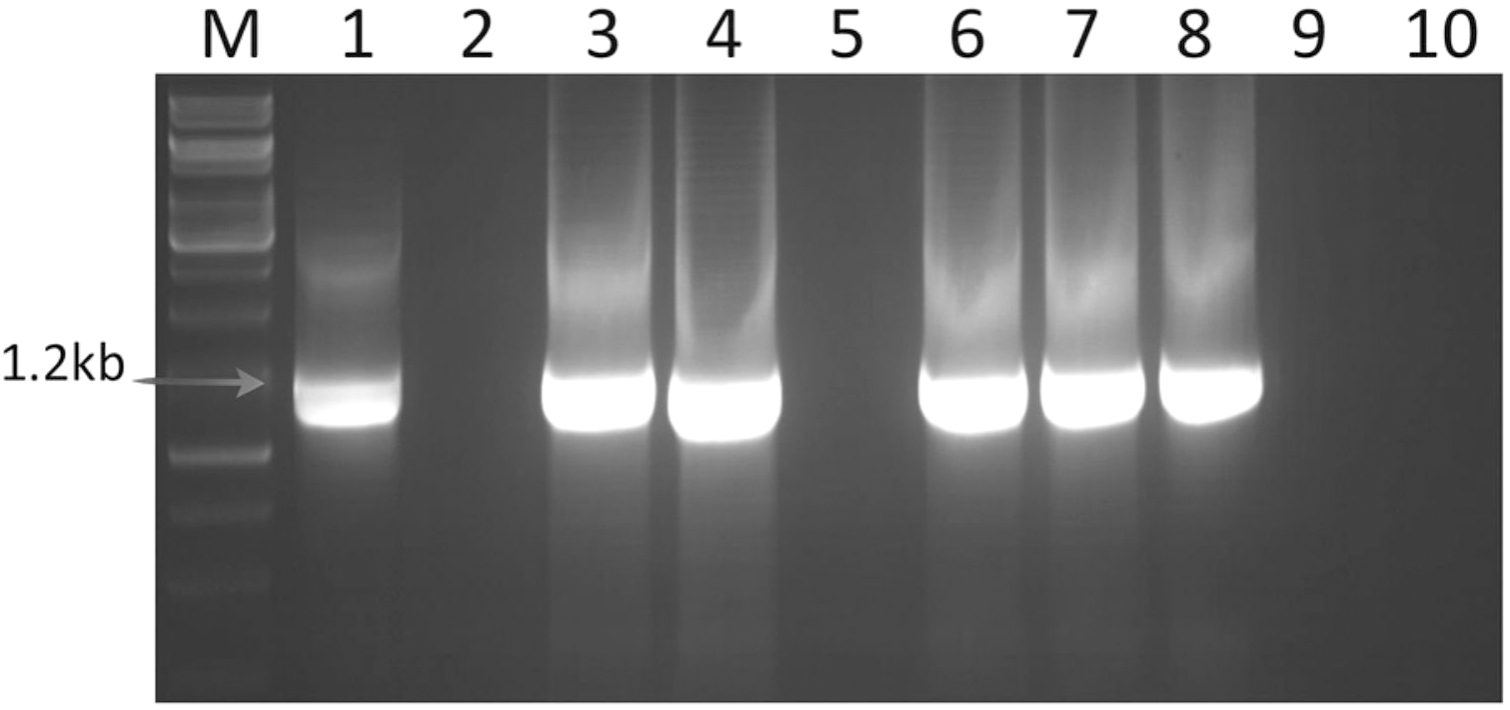
Nested PCR assay findings of phytoplasma showing gel electrophoresis image of predicted amplicons of 1.25 kb from primer pair R16F2/R16R2; Lane M, 1 kb ladder; Lane 1, Positive control (sesame phyllody phytoplasma strain, Acc. No MW622031); Lane 2, Negative control; Lane 3&4, Niger phytoplasma isolate; Lane 5, Healthy niger sample; Lane 6&7, Sesame phyllody isolate; Lane 8, *Amrasca biguttula*; Lane 9, Healthy sesame; Lane 10, *Orosius albicinctus*.

Ten symptomatic samples each of niger and sesame collected from the fields of Jabalpur showing suspected phytoplasma symptoms and the positive control of sesame phyllody maintained in a glasshouse at IARI, New Delhi, yielded ~1.8 kb amplified product in the first round PCR assays with P1/P7 universal primer pair (data not shown). The positive amplified products of the first round PCR analysis were further processed for nested PCR assays with R16F2n/R16R2 primer pair, which yielded specific amplicons of ~1.2 kb from all the symptomatic niger and sesame samples tested in the study and also from the positive control of sesame phyllody phytoplasma isolate (Acc. No. MW622031) maintained on *Catharanthus roseus* in the green house at IARI, New Delhi (**Figure 4**).

### Insect diversity and identification

Two leafhopper species, *Amrasca biguttula* and *Orosius albicinctus*, were suggested as major dominant species of leafhoppers in niger and sesame fields at JNKVV, Jabalpur, on the basis of population trapped during two years. For phytoplasma indexing, 10 collected samples of each of the insect species were subjected to a PCR assay using primer pair R16F2n/R2. Only *Amrasca biguttula* collected samples in both years tested positive for phytoplasma with the amplification of 1.25 kb in the nested PCR assay (**Figure 4**). However, no phytoplasma DNA amplification was observed in *O. albicinctus* insect samples in any of the collected years.

### Multigene sequence characteristics

**Figure 5:**
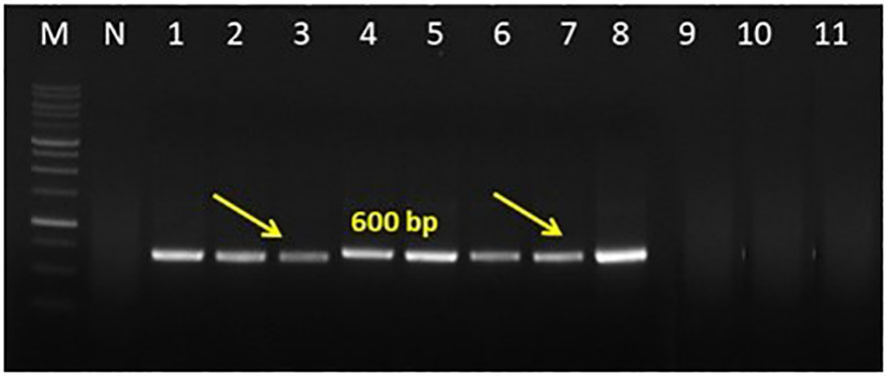
Gel electrophoresis image for nested PCR assay results of phytoplasma showing expected amplicons of 600bp from niger, sesame plant sample and leafhoppers obtained with semi nested primer pair *Sec*Afor5/*SecA*rev2; Lane M: 1kb ladder; Lane N: Negative control; Lane 1 and 2: niger phyllody; Lane 3 & 4: sesame phyllody; Lane 5 & 6: *A. biguttula*; Lane 7-8: Positive control (sesame phyllody; Acc. No. MW622031); Lane 9; *Orosius albicinctus*. Sample; Lane 10 & 11: Healthy niger and sesame samples.

**Figure 6:**
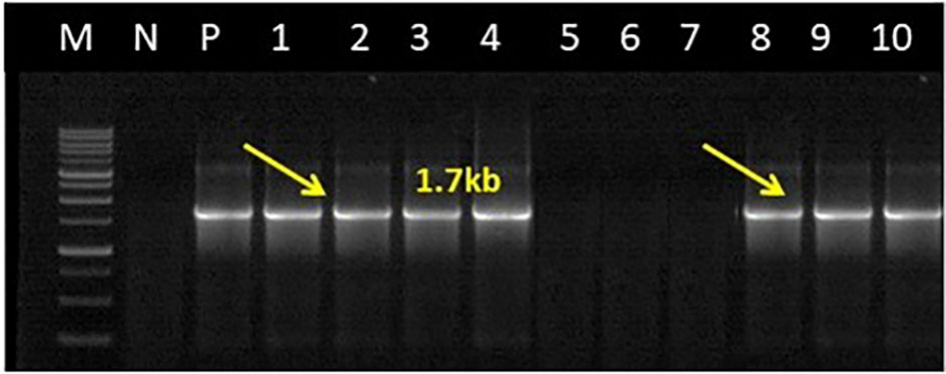
Gel electrophoresis image for nested PCR assay results of phytoplasma showing expected amplicons of 1.7kb from niger, sesame plant sample and leafhoppers obtained with semi nested primer pair secYF2(II)/secYR1(II); Lane M, 1kb ladder; Lane N, Negative control; Lane P, Positive control (sesame phyllody phytoplasma strain, Acc. No. MW622031); Lane 1 & 2, niger phyllody; Lane 3 & 4, sesame phyllody sample*;* Lane 5 & 6, Healthy niger and sesame samples; Lane 7, *Orosius albicinctus.* sample; Lane 8 & 9, *Amrasca biguttula* sample; Lane 10, Positive control.

The DNA extracted from the niger and sesame samples was analyzed with *secA* and *secY* gene-specific primers. PCR products of ~840 bp and ~600 bp with SecAfor1/SecArev3 primer pair, followed by SecAfor5/SecArev3 and ~1.7kb amplicons with direct secYF1(II)/secYR1(II) and semi-nested secYF2 (II)/secYR1(II) primer pairs were consistently amplified in the all symptomatic niger and sesame samples (**Figures 5**, **6**).

No amplifications either in the first round or nested PCR assays with a similar set of 16S rRNA, *sec*A and *sec*Y gene group-specific primers were observed in DNAs isolated from any of the niger/sesame plant samples grown in the glasshouse (negative control) and *O. albicinctus* leafhopper collected from the niger and sesame fields of JNKVV campus in both years (**Figures 4**–**6**). All the PCR amplified products from niger, sesame and insect were sequenced, analyzed and deposited in the GenBank database (**Table 2**).

### Sequence analysis

Pairwise sequence comparison of the ~1.2 kb R16F2n/R2 amplicon of the 16S rRNA gene obtained from phytoplasma isolates infecting niger (Acc. Nos. PX636087, PX636088), sesame (Acc. Nos. PX636085, PX636086), and *Amrasca biguttula* (Acc. Nos. PX636089, PX636090) revealed 100% nucleotide sequence identity among all six isolates. Further comparison with representative phytoplasma strains belonging to the 16SrII-C subgroup showed 99.76–100% sequence identity with chickpea phyllody phytoplasma (Acc. No. MT420257), faba bean phytoplasma (Acc. No.X83432), cactus witches’ broom phytoplasma (Acc. No. AJ293216), and soybean witches’ broom phytoplasma (Acc. No. HQ840717).

We also analyzed the sequence similarity index of the phytoplasma strains identified from sesame, niger, and the insect vector (*Amrasca biguttula*), under study, along with representative reference strains belonging to the 16SrII subgroups: 16SrII-A (Acc. No. L33765), 16SrII-B (Acc. No. U15442), 16SrII-C (Acc. No. AJ293216), 16SrII-D (Acc. No. LY10096), 16SrII-E (Acc. No. Y16393), and 16SrII-F (Acc. No. EF186827). Multiple sequence alignment of the sesame, niger, and *Amrasca biguttula* isolates with phytoplasma reference strains revealed 100% nucleotide sequence identity among the three isolates. Furthermore, similarity matrix analysis demonstrated that these isolates shared 100% sequence identity with the 16SrII-C subgroup reference strain (Acc. No. AJ293216). In contrast, they exhibited sequence similarity ranging from 97.7% to 99.6% with other related strains belonging to different 16SrII subgroups (**Table 3**).

**Table 3:** Nucleotide similarity matrix obtained from the alignment of sesame, niger and *Amrasca biguttula* sequences from reference phytoplasma strains.

Sr. no.	Phytoplasma isolates/subgroup reference strains	Sesamum (JB-1)	Sesamum (JB-2)	Niger (JB-5)	Niger (JB-6)	*Amrasca biguttula* (JB-9)	*Amrasca biguttula* (JB-10)	16SrII-A	16SrII-B	16SrII-C	16SrII-D	16SrII-E	16SrII-F
1	Sesamum (JB-1) PX636085	1.000											
2	Sesamum (JB-2) PX636086	1.000	1.000										
3	Niger (JB-5) PX636087	1.000	1.000	1.000									
4	Niger (JB-6) PX636088	1.000	1.000	1.000	1.000								
5	*Amrasca biguttula* (JB-9) PX636089	1.000	1.000	1.000	1.000	1.000							
6	*Amrasca biguttula* (JB-10) PX636090	1.000	1.000	1.000	1.000	1.000	1.000						
7	16SrII-A L33765	0.982	0.982	0.982	0.982	0.982	0.982	1.000					
8	16SrII-B U15442	0.991	0.991	0.991	0.991	0.991	0.991	0.980	1.000				
9	**16SrII-C AJ293216**	**0.996**	**0.996**	**0.996**	**0.996**	**0.996**	**0.996**	**0.978**	**0.987**	**1.000**			
10	16SrII-D LY10096	0.984	0.984	0.984	0.984	0.984	0.984	0.996	0.984	0.980	1.000		
11	16SrII-E Y16393	0.981	0.981	0.981	0.981	0.981	0.981	0.983	0.980	0.977	0.986	1.000	
12	16SrII-F EF186827	0.995	0.995	0.995	0.995	0.995	0.995	0.982	0.992	0.991	0.984	0.981	1.000

The bold values indicates similarity coefficient of niger, sesame and insect phytoplasma strain with references strain of 16SrII-C subgroup.

Comparison of ~840 bp partial sequences of *secA* gene of two each of niger phytoplasma isolates (Acc. No. PX531336, PX531337), sesame isolate (Acc. No. PX531334, PX531335), and *Amrasca biguttula* (Acc. Nos. PX531338, PX531339) showed 100% to 97% sequence identity with chickpea phyllody phytoplasma (Acc. No. MN728252), prima blue yellows phytoplasma (Acc. No. KJ462018) and crotalaria witches’ phytoplasma (Acc. No. KY872719) strains of 16SrII group.

Comparison of 1700 bp complete sequences of *secY* genes of two phytoplasma isolates of niger (Acc No. PX531330, PX531331), sesamum isolate (Acc. No. PX531328, PX531329) and *Amrasca biguttula* (Acc. Nos. PX531332, PX531333) had 100% to 99% sequence identity with chickpea phyllody phytoplasma (Acc. No. MN728234), crotalaria phyllody phytoplasma (Acc. No. GU004349), cactus witches’ broom phytoplasma (Acc. No. GU004323), and soyabean phyllody phytoplasma (Acc. No. GU004324) phytoplasma strains of 16SrII group.

### Phylogenetic relationship

**Figure 7:**
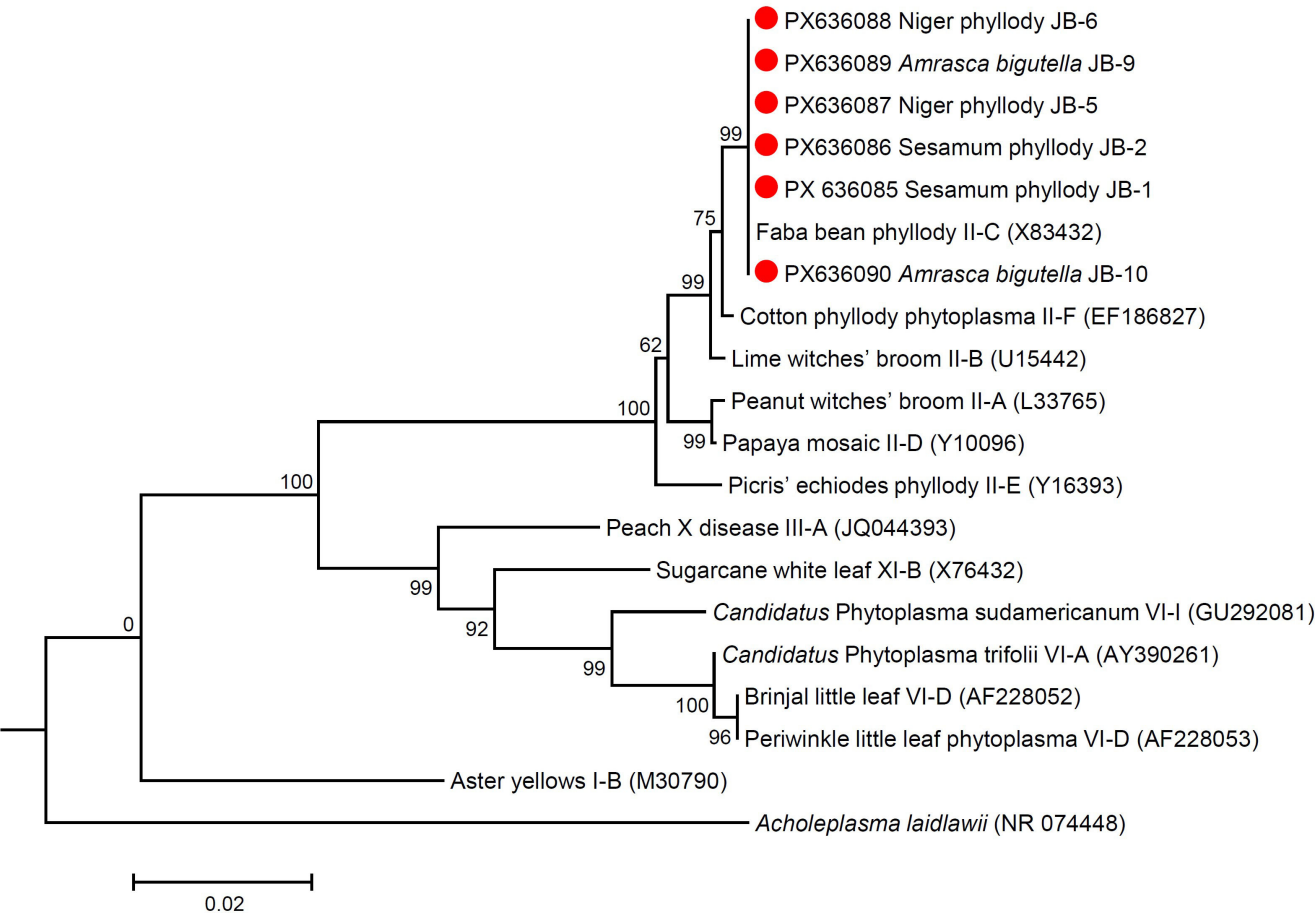
Phylogenetic tree of 16S rRNA gene sequences constructed by the neighbor joining method showing the relationships among niger, sesame and insect phytoplasma isolates with reference phytoplasma strains. Numbers on branches are bootstrap values obtained for 1000 bootstrap replicates. The bar represents a phylogenetic distance of 0.02.

**Figure 8:**
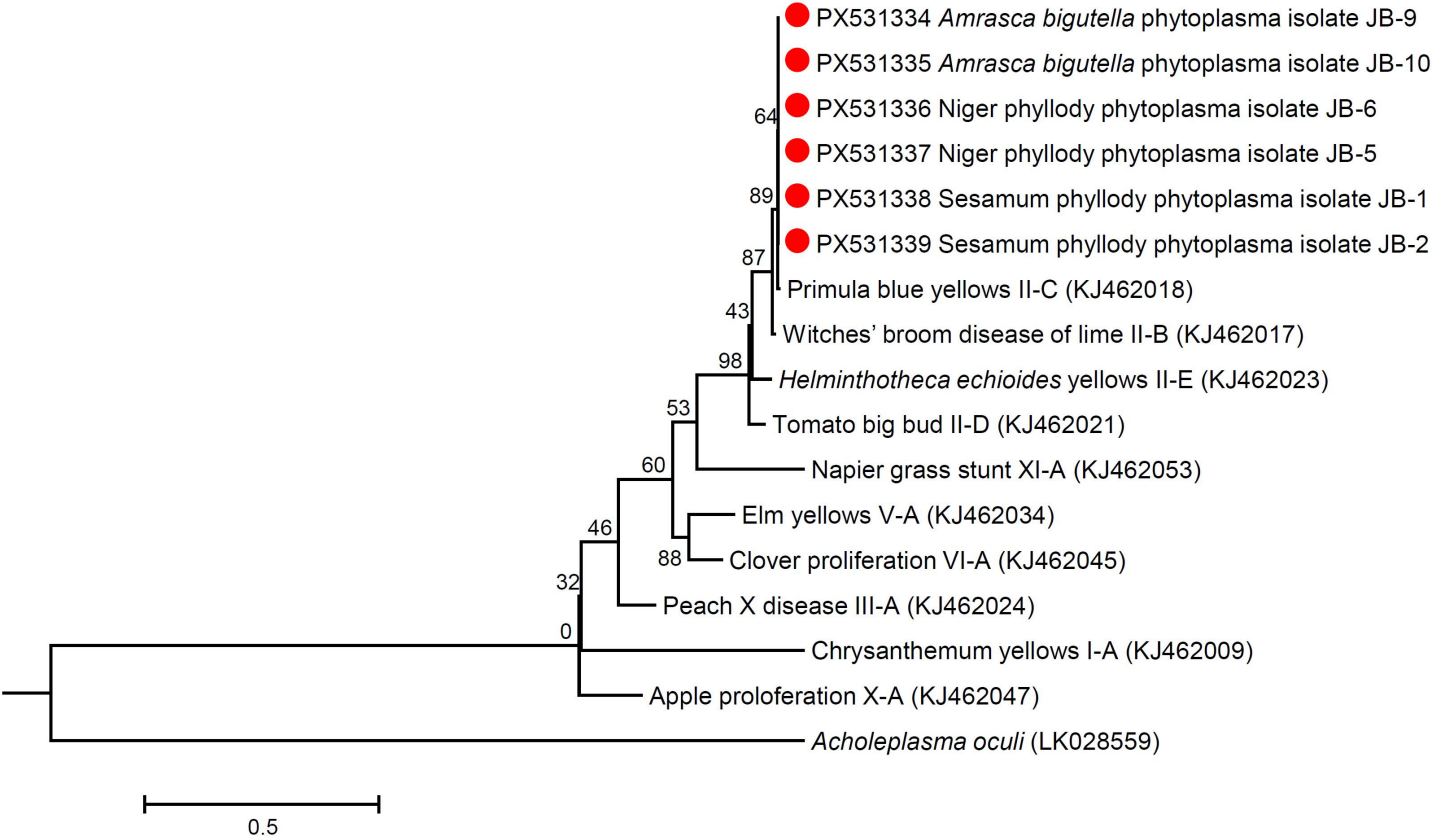
Phylogenetic tree of *sec*A gene sequences constructed by neighbor-joining method showing the relationships among niger, sesame and insect phytoplasma isolates with reference phytoplasma strains. The tree was rooted with *Acholeplasma oculi*. Numbers on branches are bootstrap values obtained for 1000 bootstrap replicates. The bar represents a phylogenetic distance of 0.5.

**Figure 9:**
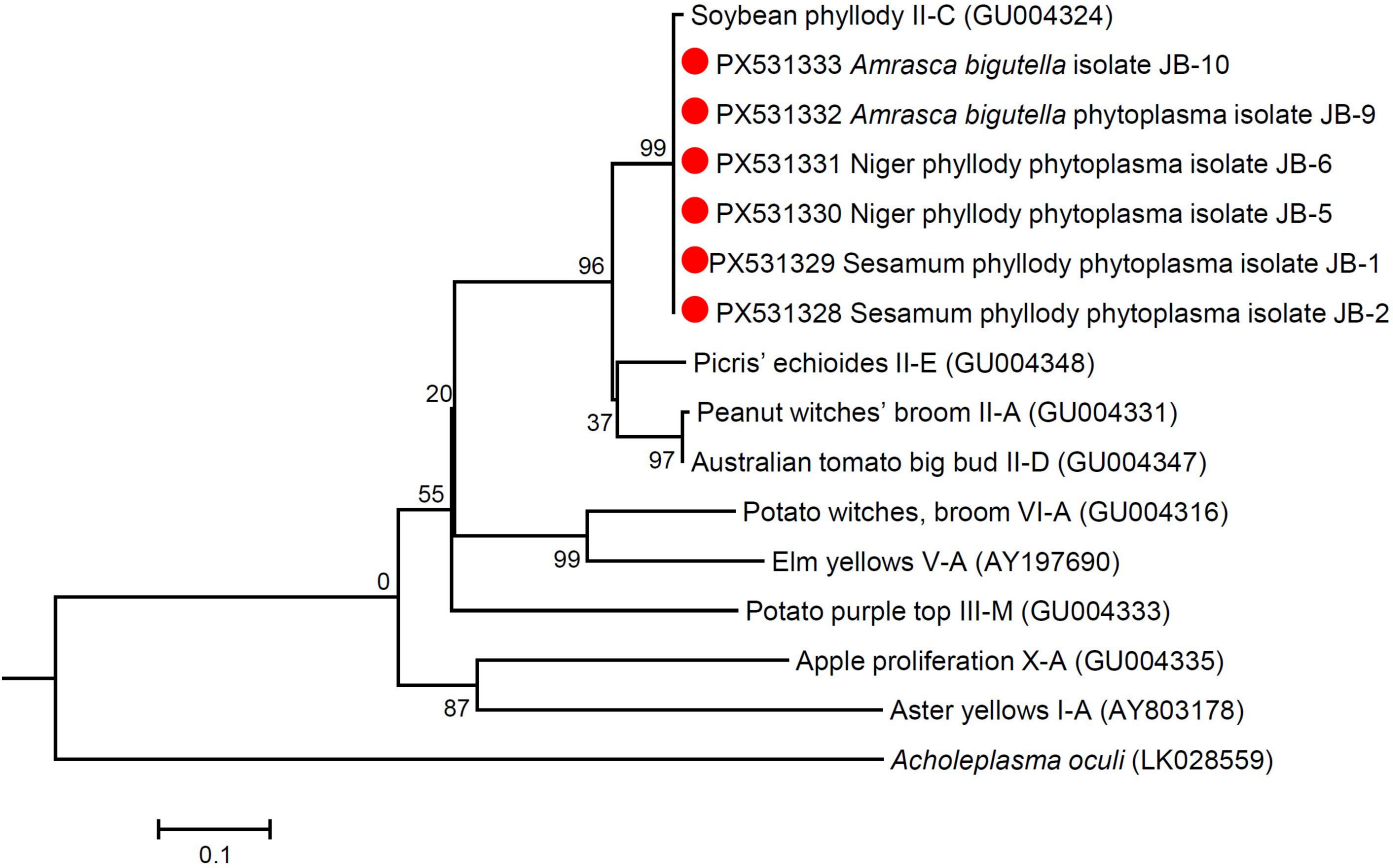
Phylogenetic tree of *secY* gene sequences constructed by the neighbor-joining method showing the relationships among niger, sesame and insect phytoplasma isolates with reference phytoplasma strains. The tree was rooted with *Acholeplasma oculi*. Numbers on branches are bootstrap values obtained for 1000 bootstrap replicates. The bar represents a phylogenetic distance of 0.1.

Phylogenetic analysis of the 16S rRNA sequences of niger, sesame and *Amrasca biguttula* phytoplasma isolates with those of submitted sequences in GenBank revealed their close phylogenetic relationship with members of the peanut witches’ broom (16SrII) group. It is evident from the results that the niger, sesame and insect phytoplasma isolates were clustered in a sub-clade with 16SrII phytoplasma-related strains of 16SrII-C subgroup (*Ca*. P. asiaticum) in the phylogeny tree (**Figure 7**; **Supplementary Table 1**). Similar results were obtained with the phylogenetic comparison analysis with *sec*A and *sec*Y gene sequences of niger, sesame and insect phytoplasma isolates when compared with those of reference strains of phytoplasma sequences in GenBank (**Figures 8**, **9**; **Supplementary Table 1**). The phylogenetic analysis of 16S rRNA, *sec*A and secY gene sequences confirmed the association of peanut witches’ broom (16SrII) group with symptomatic niger, sesame and *Amrasca biguttula* samples in the present study.

### *In silico* RFLP analysis

**Figure 10:**
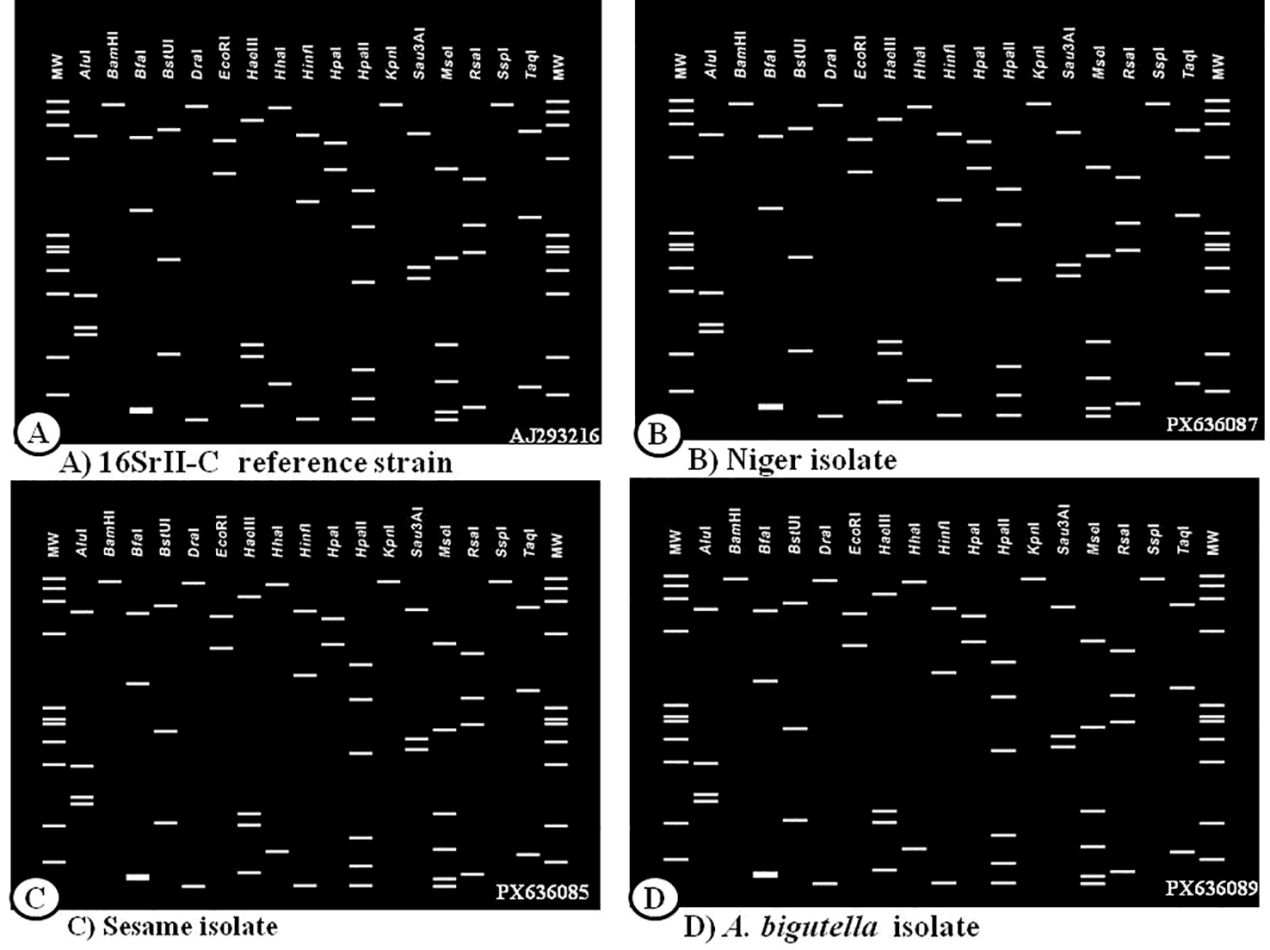
Comparison of virtual RFLP pattern derived from in silico digestion of ~1.25kb *16SrRNA* sequences of reference phytoplasma subgroup with 17 different restriction endonucleases using *i*phyclassifier program **(a)** 16SrII-C reference strain (Acc. No. AJ293216), **(b)** niger isolate (Acc. No. PX636087), **(c)** sesame isolate (Acc. No. PX636085); **(d)** *Amrasca biguttula* isolate (Acc. No PX636089).

The virtual RFLP analysis of the F2nR2 region of 16S rRNA gene of niger, sesame and insect phytoplasma isolates was compared for the 16Sr group and subgroup assignment using *iPhy*Classifier online tool. Comparison of the restriction site maps revealed that six isolates produced a similar virtual RFLP profile identical to the reference strain for 16SrII-C sub-group (Acc. No. AJ293216) (**Figures 10A–D**) with the similarity coefficient value of 0.99. On the basis of restriction profiles, the sesame, niger and leafhopper phytoplasma isolates in the present study were classified under the peanut witches’ broom group under 16SrII-C subgroup (*Candidatus* Phytoplasma asistaicum).

## Discussion

Niger (*Guizotia abyssinica*) is a vital oilseed crop cultivated predominantly on marginal lands under rainfed conditions across Asia and Africa. India, as the global leader in niger cultivation area, faces significant yield constraints due to biotic stresses, with phytoplasma infections representing an emerging threat to sustainable production (Kolte, 1985; Gupta et al., 2023; Rao, 2021; Tiwari et al., 2023). Similarly, sesame (*Sesamum indicum* L.), another economically crucial oilseed crop, is highly susceptible to phytoplasma infections, which cause devastating diseases like phyllody and witches’ broom, leading to substantial yield losses worldwide (Rao et al., 2017). The recent expansion of phytoplasma diseases across these crops and other oilseed agro-ecosystems underscores their escalating impact on agricultural productivity and food security (Rao, 2021). The co-cultivation of niger and sesame in many regions, including central India, creates a conducive environment for the spread of these pathogens, complicating disease management efforts.

Our study established a novel association between the 16SrII-C subgroup (‘*Ca.* P. asiaticum’) and niger phyllody in India, specifically associated with leaf yellowing and flower malformation symptoms in Jabalpur. This finding expands the known phytoplasma diversity affecting niger beyond the previously reported 16SrI-B and 16SrII-D subgroups from India and Iran (Muttappagol et al., 2022; Vaali et al., 2011). The concurrent identification of sesame as a host for the identical 16SrII-C strain is particularly significant, as sesame has been reported as a host for multiple phytoplasma groups (16SrI, II, VI) across different regions (Rao and Nabi, 2015; Singh et al., 2016). This multi-host compatibility aligns with recent findings by Tiwari et al. (2023), who demonstrated the ability of 16SrII group phytoplasmas to infect multiple crop species within the same agricultural landscape.

Our multilocus sequence analysis using *sec*A and *sec*Y genes provided robust confirmation of the *Ca.* P. asistaicum related strains (16SrII-C subgroup) identity across all symptomatic samples. This approach aligns with recent recommendations for phytoplasma taxonomy, as the 16S rRNA gene often lacks sufficient polymorphism for reliable strain differentiation (Wei and Zhao, 2022). The consistent amplification of these genetic markers across plant and insect samples validates their utility for precise phytoplasma characterization, which emphasized the necessity of multilocus genotyping for accurate phytoplasma classification and evolutionary studies (Bertaccini et al., 2022).

The detection of identical 16SrII-C strains in the leafhopper *A. biguttula* confirms possibility of its vector status in this pathosystem, expanding its host range for various phytoplasma strains across different cropping systems (Bandakkanavara, 2021). The coexistence of infected niger and sesame creates a perennial reservoir that facilitates pathogen persistence and spread, consistent with the “green bridge” effect in other phytoplasma pathosystems. The widespread cultivation of these crops across Madhya Pradesh, combined with the polyphagous nature of the vector, establishes conditions conducive to rapid disease dissemination across the region.

The emergence of 16SrII-C phytoplasma (*Ca*. P. asiaticum) in niger from India represents a significant shift in the disease epidemiology. While the 16SrII-D subgroup has been predominant in previous reports from India, the identification of 16SrII-C suggests either a recent introduction or diversification of phytoplasma strains in the region. Similar patterns of phytoplasma subgroup replacement have been observed in other crops, including sesame and sugarcane, potentially linked to changing agricultural practices and climate conditions (Reddy et al., 2021; Rao, 2021).

The identification of an identical ‘*Ca*. P. asiaticum’ strain (16SrII-C) in symptomatic niger, sesame, and the leafhopper *A. biguttula* confirms a functional and efficient transmission cycle within the agro-ecosystem. The polyphagous nature of *A. biguttula*, which has been documented to feed on multiple crops and weed species, makes it a highly effective vector for disseminating the pathogen across the landscape (Bandakkanavara, 2021). The simultaneous cultivation of niger and sesame, which often have overlapping growing seasons, creates a persistent “green bridge” effect. This allows the phytoplasma to perpetuate and amplify, as the vector can sequentially acquire the pathogen from an infected niger plant and inoculate it into a healthy sesame plant, and vice versa, throughout the cropping season (Kumari et al., 2019). However, in the present study, only a small sampling size and number of insects was tested for phytoplasma selection and does not warrant a claim the *Amrasca biguttula* as natural vector. This needs further study confirmation through transmission assays in different cropping season which is under progress. Beyond cultivated crops, the role of alternative weed hosts in the epidemiology of 16SrII group phytoplasmas is also recognized (Rao, 2021; Yadav et al., 2015). Furthermore, changing climatic conditions, particularly rising temperatures, may be extending the active period of leafhopper vectors and accelerating the pathogen’s incubation period within them, potentially leading to more rapid and widespread epidemics (Hossain et al., 2024). The widespread cultivation of niger and sesame across Madhya Pradesh, combined with the lack of resistant varieties, establishes a high-risk scenario for the large-scale dissemination of this 16SrII-C strain, threatening not only these two crops but also other economically important hosts in the region. The observational trends of emergence of a 16SrII-C phytoplasma strain within this dual-host cropping system, coupled with an efficient leafhopper vector, necessitates an integrated management approach. Our findings underscore the urgency of implementing coordinated strategies, including: (1) the systematic screening of niger and sesame germplasm to identify and introgress resistant traits, as successfully demonstrated in other phytoplasma pathosystems (2) the establishment of robust vector surveillance and area-wide control programs targeting *A. biguttula*, focusing on critical transmission windows to disrupt the pathogen’s life cycle; and (3) comprehensive ecological management through the identification and control of alternative weed hosts to eliminate reservoir-based inoculum and break the disease cycle, as advocated in recent ecological studies (Yadav et al., 2022). This multifaceted strategy is essential to mitigate the escalating threat to oilseed production in the region.

## Conclusion

This research provides the first molecular evidence of a 16SrII-C subgroup phytoplasma (*‘Ca.* P. asiaticum’) associated with niger phyllody in the Jabalpur region, while simultaneously identifying sesame as a natural reservoir for the similar phytoplasma strain. The consistent detection of this phytoplasma strain across multiple symptomatic plant samples confirms its role in the observed disease symptoms, including characteristic leaf yellowing and phyllody manifestations in both the crop species. Our investigation also establishes the leafhopper *A. biguttula* as a putative vector in this pathosystem, representing the first documented evidence of this specific insect-phytoplasma association within Madhya Pradesh’s oilseed cultivation system. The molecular detection and observational trends of identical phytoplasma strains in field-collected leafhoppers and symptomatic plants confirms *A*. *biguttula*’s possible role in pathogen transmission, completing the epidemiological triangle in this emerging disease complex. The application of multilocus sequence analysis using *sec*A and *sec*Y genes proved essential for precise strain differentiation, demonstrating significant advantages over single-gene characterization. This comprehensive molecular approach enabled reliable cross-host verification of pathogen identity. It facilitated accurate phylogenetic placement of the identified strain within the 16SrII group, confirming its classification as a 16SrII-C subgroup phytoplasma. Based on these findings, future research should prioritize controlled transmission assays to quantitatively evaluate *A. biguttula’s* vector efficiency and host preferences. Additionally, epidemiological modeling incorporating environmental parameters and cropping patterns will be crucial for predicting disease spread dynamics. These investigations should be complemented by expanded resistance screening of niger and sesame germplasm, which will provide the scientific foundation for developing integrated management strategies against this emerging phytoplasma disease complex in India’s oilseed crop production systems.

## Data Availability

The datasets presented in this study can be found in online repositories. The names of the repository/repositories and accession number(s) can be found in the article/**Supplementary Material**.
