# Supplementary material for: Multigene analysis reveals ‘Candidatus Phytoplasma asiaticum’ (16SrII-C) association with niger and sesame phyllody in Madhya Pradesh, Central India and identification of a putative vector
Source: Front Plant Sci. 2026 Apr 13;17:1768105. doi: 10.3389/fpls.2026.1768105 (PMC13111267; doi:10.3389/fpls.2026.1768105)
Supplement: Supplementary file 1 [file Table1.doc]

**Supplementary Table 1.** Phytoplasma gene sequences (16S rRNA, *sec*A*, sec*Y) retrieved from GenBank utilized for deriving phylogenetic relationships of niger phyllody, sesame phyllody and *Amrasca bigutella* phytoplasma isolates in the present study

| **Genbank accession no** | **Phytoplasma strain** | **Subgroup** | **Host** | **Country** |
| --- | --- | --- | --- | --- |
| **16S rRNA gene** | | | | |
| PX636088 | Niger phyllody JB-6 | 16SrII-C | *Guizotia abyssinica* | India (present study) |
| PX636087 | Niger phyllody JB-5 | 16SrII-C | *Guizotia abyssinica* | India (present study) |
| PX636086 | Sesamum phyllody JB-2 | 16SrII-C | *Sesamum indicum* | India (present study) |
| PX636085 | Sesamum phyllody JB-1 | 16SrII-C | *Sesamum indicum* | India (present study) |
| PX636089 | *Amrasca bigutella* JB-9 | 16SrII-C | *Sesamum indicum* | India (present study) |
| PX636090 | *Amrasca bigutella* JB-10 | 16SrII-C | *Guizotia abyssinica* | India (present study) |
| X83432 | Faba bean phyllody | 16SrII-C | *Vicia faba* | Germany |
| EF186827 | Cotton phyllody phytoplasma | 16SrII-F | Gossypium | Beltsville, USA |
| L33765 | Peanut witches-broom | 16SrII-A | *Catharanthus roseus* | Beltsville, USA |
| Y16393 | *Picris echiodes* phyllody | 16SrII-E | *Picris echioides* | Italy |
| JQ044393 | Peach X disease | 16SrIII-A | *Prunus persica* | Beltsville, USA |
| GU292081 | Clover proliferation | 16SrVI | *Passiflora edulis* | Beltsville, USA |
| M30790 | Aster yellows | 16SrI-B | *Oenothera hookeri* | East Lansing, U.S |
| AY390261 | Clover proliferation | 16SrVI-A | *Trifolium hybridum* | Canada: Alberta |
| AF228052 | Brinjal little leaf | 16SrVI-D | *Solanum melongena* | India: Karnataka |
| AF228053 | Periwinkle little leaf phytoplasma | 16SrVI-D | *Catharanthus roseus* | India: Karnataka |
| NR074448 |  | Outgroup | *Acholeplasma laidlawii* |  |
| ***sec*A gene** | | | | |
| PX531334 | Sesamum phyllody JB-1 | 16SrII-C | *Sesamum indicum* | India (present study) |
| PX531335 | Sesamum phyllody JB-2 | 16SrII-C | *Sesamum indicum* | India (present study) |
| PX531336 | Niger phyllody JB-5 | 16SrII-C | *Guizotia abyssinica* | India (present study) |
| PX531337 | Niger phyllody JB-6 | 16SrII-C | *Guizotia abyssinica* | India (present study) |
| PX531338 | *Amrasca bigutella* JB-9 | 16SrII-C | *Sesamum indicum* | India (present study) |
| PX531339 | *Amrasca bigutella* JB-10 | 16SrII-C | *Guizotia abyssinica* | India (present study) |
| KJ462018 | Primula blue yellows | 16SrII | Primula blue | UK |
| KJ462017 | Witches' broom disease of lime | 16SrII-B | *Citrus aurantifolia* | Oman |
| KJ462023 | Helminthotheca echioides yellows | 16SrII-E | Bristly oxtongue | Italy |
| KJ462021 | Tomato big bud | 16SrII-D | tomato | Australia |
| KJ462053 | Napier grass stunt | 16SrXI -A | *Pennisetum purpureum* | Uganda |
| KJ462034 | Elm yellows | 16SrV -A | Elm | USA |
| KJ462045 | Clover proliferation | 16SrVI-A | clover | Canada |
| KJ462024 | Peach X disease | 16SrIII -A | peach | Canada |
| KJ462009 | Chrysanthemum yellows | 16SrI -A | *Chrysanthemum frutescens* | Germany |
| KJ462047 | Apple proliferation | 16SrX-A | Apple cv. Golden Delicious | Germany |
| LK028559 |  | Outgroup | *Acholeplasma oculi* | Germany |
| ***sec*Y gene** | | | | |
| PX531328 | Sesamum phyllody JB-1 | 16SrII-C | *Sesamum indicum* | India (present study) |
| PX531329 | Sesamum phyllody JB-2 | 16SrII-C | *Sesamum indicum* | India (present study) |
| PX531330 | Niger phyllody JB-5 | 16SrII-C | *Guizotia abyssinica* | India (present study) |
| PX531331 | Niger phyllody JB-6 | 16SrII-C | *Guizotia abyssinica* | India (present study) |
| PX531332 | *Amrasca bigutella* JB-9 | 16SrII-C | *Sesamum indicum* | India (present study) |
| PX531333 | *Amrasca bigutella* JB-10 | 16SrII-C | *Guizotia abyssinica* | India (present study) |
| GU004324 | Soybean phylSoyabean phyllody | 16SrII-C | Soybean | Thailand |
| GU004348 | *Picris echioides* | 16SrI-E | *Pichris echioides* | Italy |
| GU004331 | Peanut witches' broom | 16SrII-A | Peanut | Taiwan |
| GU004347 | Australian tomato big bud | 16SrII-D | Tomato | Australia |
| GU004316 | Potato witches' broom | 16SrVI-A | Potato | Canada |
| AY197690 | Elm yellows | 16SrV-A | *Ulmus americana* | Beltsville, USA |
| GU004333 | Potato purple top | 16SrIII-M | Potato | USA |
| GU004335 | Apple proliferation | 16SrX-A | Apple | Italy |
| AY803178 | Aster yellows | 16SrI-A | Tomato | USA |
| LK028559 |  | Outgroup | *Acholeplasma oculi* | Germany |
